# Acute and chronic changes in porcine rete mirabile after embolization with the Menox system: angiographic and histopathological analyses

**DOI:** 10.1590/0100-3984.2022.0054

**Published:** 2023

**Authors:** Ricardo Miguel Costa de Freitas, Mauricio Ricardo Moreira da Silva Filho, Alessandro Rodrigo Belon, Brasil Chian Ping Jeng, Denilson Mayrink, José Guilherme Mendes Pereira Caldas

**Affiliations:** 1 Department of Radiology, Faculdade de Medicina da Universidade de São Paulo (FMUSP), São Paulo, SP, Brazil; 2 Instituto do Câncer do Estado de São Paulo (Icesp), São Paulo, SP, Brazil; 3 Department of Experimental Surgery, Faculdade de Medicina da Universidade de São Paulo (FMUSP), São Paulo, SP, Brazil; 4 Department of Neurosurgery, Faculdade de Medicina da Universidade de São Paulo (FMUSP), São Paulo, SP, Brazil; 5 Department of Pathology, Diagnostika Laboratory, São Paulo, SP, Brazil

**Keywords:** Skull base/blood supply, Embolization, therapeutic, Dimethyl sulfoxide/therapeutic use, Polyvinyls/therapeutic use, Swine, Models, animal, Base do crânio/irrigação sanguínea, Embolização, terapêutica, Dimetil sulfóxido/uso terapêutico, Polivinil/uso terapêutico, Suínos, Modelos animais

## Abstract

**Objective:**

To evaluate acute and chronic changes seen on angiographic and histopathological studies of porcine rete mirabile, comparing those treated with the Menox liquid embolic system (LES) and those treated with the Onyx LES.

**Materials and Methods:**

Five pigs, each weighing approximately 35 kg, were submitted to rete mirabile embolization under general anesthesia and fluoroscopic guidance, with the Menox LES or Onyx LES. Four animals were treated with the Menox LES and underwent cerebral angiography, followed by euthanasia, at 1, 30, 60, and 90 days after embolization. One animal was treated with the Onyx LES underwent the same procedures at 30 days after embolization. In a subsequent histopathological analysis, we compared the Menox LES and Onyx LES in terms of the acute and chronic changes observed.

**Results:**

We observed no significant changes in blood pressure, heart rate, or electrocardiographic parameters that could be attributed to the super-selective infusion of dimethyl sulfoxide or the Menox embolic agent. Fluoroscopy showed adequate material opacity, appropriate progression to the center of the rete mirabile and complete unilateral embolization. Microcatheters were uneventfully detached from the embolized nidus. We observed mild to moderate intravascular and extravascular inflammatory responses, without histological evidence of necrotizing arteritis. There were no adverse neurovascular events.

**Conclusion:**

The Menox LES appears to be safe and effective, as well as being apparently equivalent to the Onyx LES in terms of the postprocedure angiographic and histopathological findings.

## Abstract (pt)

**Objetivo:**

Avaliar as alterações angiográficas e histopatológicas agudas e crônicas em *rete mirabile* suína tratadas com o Menox *liquid embolic system* (LES) e comparar essas alterações com a embolização com Onyx LES.

**Materiais e Métodos:**

A embolização da *rete mirabile* com Menox LES e Onyx LES foi realizada em cinco suínos pesando cerca de 35 kg sob anestesia geral e orientação fluoroscópica. Quatro animais tratados com Menox LES foram submetidos a angiografia cerebral seguida de eutanásia após 1, 30, 60 e 90 dias e um animal tratado com Onix LES foi submetido ao mesmo procedimento após 30 dias. A análise histopatológica subsequente para alterações agudas e crônicas avaliou o desempenho do Menox LES comparado ao Onyx LES.

**Resultados:**

Não foram observadas alterações significativas atribuíveis à infusão superseletiva de dimetilsulfóxido ou Menox nos parâmetros de pressão arterial, frequência cardíaca ou eletrocardiograma. A fluoroscopia mostrou opacidade adequada do material, progressão adequada para o centro da *rete mirabile* e embolização unilateral completa. Os microcateteres foram retirados do *nidus* embolizado sem complicações. Observou-se resposta inflamatória intravascular e extravascular leve a moderada, sem indício histológico de arterite necrosante. Nenhum dos casos apresentou eventos neurovasculares adversos.

**Conclusão:**

A injeção de Menox LES mostrou-se segura e eficaz, além de ser equivalente ao Onyx LES em relação aos achados angiográficos e histopatológicos pós-procedimento.

## INTRODUCTION

Preoperative endovascular embolization is an effective neoadjuvant treatment that decreases the size of large cerebral arteriovenous malformations (AVMs), thereby reducing operative time, blood loss, morbidity, and mortality^(1-4)^. Recent technological advances, such as the development of liquid embolic agents (LEAs), have increased embolization success rates. The most widely used LEAs are non-adhesive copolymers of ethylene-vinyl alcohol (EVOH), including Onyx (Covidien, Irvine, CA, USA) and the newly developed Menox (Meril Life Sciences, Gujarat, India), both of which have been evaluated in various studies^(5-9)^. The purpose of this study was to evaluate the feasibility, reproducibility, and safety of the Menox liquid embolic system (LES), in comparison with the Onyx LES, in a porcine model of AVM embolization.

## MATERIALS AND METHODS

### Test materials

The Menox LES employs a non-adhesive liquid embolic material composed of EVOH copolymer dissolved in dimethyl sulfoxide (DMSO), and suspended micronized tantalum powder to provide contrast for visualization under fluoroscopy. A DMSO-compatible microcatheter is used in order to access the embolization site. The DMSO is injected to fill the dead space of the microcatheter because it prevents unintentional precipitation of the Menox LEA, which begins immediately after it comes into contact with water, saline solution, or blood. The Menox LEA is then delivered by slow, controlled injection under fluoroscopic guidance. The DMSO dissipates into the blood, causing the EVOH copolymer and suspended tantalum to precipitate *in situ* into a spongy, cohesive embolus. The Menox LES comprises a 1.5-mL vial of Menox LEA, a 1.5-mL vial of DMSO, and three 1-mL Menox delivery DMSO-compatible Luer lock syringes. The control was the Onyx LES, which employs a minimally adhesive LEA, comprising a 1.5-mL vial of Onyx LEA, a 1.5-mL vial of DMSO, and three 1-mL Onyx delivery syringes. A 5F guiding catheter, a 5F sheath (with a 20G access needle), and sterile DMSO-compatible microcatheters (Marathon 1.5 F; ev3, Inc., Plymouth, MN, USA) were also employed.

### Animal model

**Figure 1:**
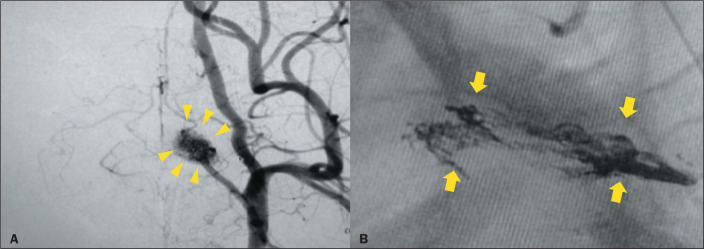
Fluoroscopy of (A) a normal RM (arrowheads), and (B) an embolized RM (arrows).

In pigs, the rete mirabile (RM), as depicted in Figure 1A, is a microvascular network that structurally resembles the human cerebral AVM nidus^(10-12)^. It is located at the skull base, within the cavernous sinuses, between the ascending pharyngeal artery and the internal carotid artery. The right and left RM communicate across the midline. The experimental protocol employed in the present study was approved by the local animal research ethics committee. We selected 4-month-old, pathogen-free domestic pigs (*Sus scrofa domesticus*; Agroceres, São Paulo, Brazil), weighing 32-34 kg, that had been physically assessed under the supervision of the facility veterinarian. In accordance with the sufficiency criterion and the financial capability of the study to meet its main objectives, the sample comprised five pigs (two males and three females), group-housed and maintained on a standard laboratory diet under the supervision of the facility veterinarian. The animals were allowed to acclimate to the environment for at least 33 days prior to the experiments. The animals were randomly divided into two groups: the study group (pigs 1, 2, and 3), in which the animals were assessed to identify acute and chronic changes on angiography and histopathology after Menox LES embolization; and the comparison group (pigs 4 and 5), in which the animals were assessed, also by angiography and histopathology, to identify differences between the Menox LES and the Onyx LES, in terms of the postembolization effects.

### Endpoints

This study endpoint was complete unilateral occlusion of the RM with no technical complications or major adverse neurovascular, hemodynamic or thromboembolic events, such as stroke, transient ischemic attack, reversible ischemic neurological deficit, thrombosis, and death.

### Procedural and technical details

All procedures were performed in the interventional radiology department of a university hospital. The animals were sedated, intubated under general anesthesia, and positioned in the prone position on the bed of a cone-beam computed tomography unit (Innova 4100; GE Healthcare, Waukesha, WI, USA). Cardiac and respiratory parameters were monitored during and after the procedure. The core temperature was maintained at 37-39°C. The embolization procedures were performed under sterile conditions. Through a 20G access needle, a 5F sheath was introduced into the right common femoral artery. Under fluoroscopic guidance, a 5F guiding catheter that had been flushed continuously with saline was inserted into the right or left common carotid artery. Positioning was confirmed in three-dimensional images. Control angiography was performed, after which a DMSO-compatible microcatheter was advanced into the proximal RM until met with resistance and then wedged in place. Before the injection, the Menox LEA was shaken for at least 20 min on a Vortex mixer. Appropriate 1-mL DMSO-compatible syringes were used in order to inject the DMSO and Menox LEA. The contrast was flushed from the microcatheter hub with 10 mL of saline. The Menox LEA was injected at a steady rate of 0.16 mL/min (0.25 mL/90 s) to displace the DMSO. The injection and reflux of Menox LEA into the RM was controlled based on observation of the filling pattern: all portions of the ipsilateral RM, including the central portion, were filled, and injection would stop when it reached the contralateral RM (Figure 1B). The volume of Menox LEA reflux considered acceptable was 1 cm. The microcatheter was retrieved using a few centimeters of traction. Obliteration and any intracranial leaks of the Menox LEA were assessed with angiography at the end of each embolization procedure. The control animal was embolized with the Onyx LEA in the same manner detailed above.

### Outcome measures

During the procedure, we used fluoroscopy to assess the ease of delivery through the microcatheter, controllability (ability to define the start and end points of embolization, radiopacity, injection speed, and volume), extent/depth of penetration into the RM, duration of occlusion of the RM (long-term follow-up), and microcatheter adherence to the artery.

After the procedure, all of the animals were followed at the animal facility, and neurologic assessment was performed regularly. Pain signs such as vocalization, lethargy, limping or loss of appetite were noted. At the end of the clinical follow-up period, the animals were euthanized. The animals were sedated before receiving lethal doses of potassium chloride to induce cardiac arrest. In the study group, one animal was euthanized 24 h after the procedure, one at 60 days after and one at 90 days after. In the comparison group, the animal treated with the Menox LES and the animal treated with the Onyx LES were both euthanized at 30 days after the procedure.

After the animals had been euthanized, each RM was carefully exposed and dissected from the cavernous sinus. Tissues were fixed in 10% neutral buffered formalin at 37°C, processed into paraffin, sectioned at 5 µm, and stained with hematoxylin and eosin (H&E) and elastic Van Gieson stains. An experienced neuropathologist evaluated histopathological changes in the embolized RM. The specimen underwent gross examination to identify postembolization changes, including changes in texture or consistency, as well as thrombosis, extravasation, an inflammatory or granulomatous response, and fibrosis.

None of the animals had any significant clinical abnormalities or experienced any significant adverse events before, during, or after the procedure. In all cases, the RM was assessed by angiography, which confirmed the presence and distribution of the Menox or Onyx LEA. Hemodynamic monitoring showed no instability during any of the procedures. We observed no significant changes in blood pressure, heart rate, or electrocardiographic parameters that could be attributable to the super-selective infusion of DMSO or the Menox LEA. No thromboembolic events or intracranial/extracranial extravasation of the Menox LEA were observed on angiography, and fluoroscopy showed adequate LEA opacity. In all five animals, we observed complete unilateral RM embolization with appropriate LEA progression to the center of the RM. The microcatheters were successfully and easily detached from the embolized nidus. After the procedure, all of the animals awoke with no deficits, and there were no adverse neurovascular events before, during, or after the procedure. After euthanasia, craniotomy with dissection of the RM and brain revealed a change in the color of the RM, with no signs of thrombosis in the skull base blood vessels or extra-RM extravasation.

The animals in the study group (pigs 1-3) were assessed for acute and chronic changes by angiography and histopathology after Menox LES embolization.

**Figure 2:**
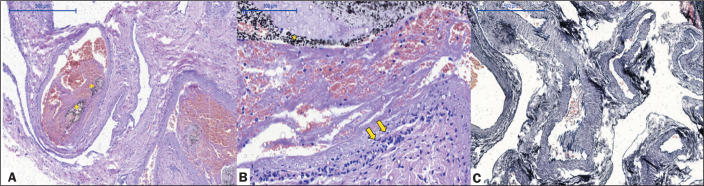
Acute histopathological findings at 24 h after Menox LES embolization of the RM, showing intraluminal LEA (asterisks), with a mild perivascular inflammatory reaction, a predominance of mononuclear cells (arrows), and necrotic foci in the tunica media of the vessels, with no giant cells or LEA extravasation (H&E staining; magnification, ×5 in A and ×20 in B), as well as elastic fibers within the vessel walls (Van Gieson staining in C; magnification, ×10).

Pig 1 (male, 34 kg) - euthanized at 24 h after the procedure: Menox LES RM embolization was performed for 20 min. There was occlusion of the right RM, without complications during microcatheter retrieval. Histopathological analysis showed a mild perivascular inflammatory reaction, with a predominance of mononuclear cells and necrotic foci in the tunica media of the vessel, with no giant cells or extravasation of the LEA (Figure 2).

**Figure 3:**
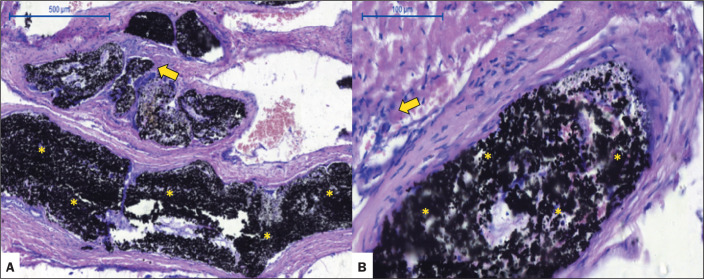
Chronic histopathological findings at 60 days after Menox LES embolization of the RM, showing intraluminal LEA (asterisks), with some microscopic extravasation of the LEA, as well as mild intravascular and extravascular inflammatory reactions, with a predominance of giant cells and few lymphocytes (arrows, H&E staining; magnification, ×5 in A and ×20 in B).

Pig 2 (male, 34 kg) - euthanized at 60 days after the procedure: Menox LES RM embolization was performed for 10 min. There was complete occlusion of the right and middle RM, without complications during microcatheter retrieval. Angiography performed at 60 days after the procedure showed LEA stability and occlusion of the right and middle RM. Histopathological analysis showed some microscopic extravasation of the LEA and mild intravascular and extravascular inflammatory reactions, with a predominance of giant cells and few lymphocytes (Figure 3).

**Figure 4:**
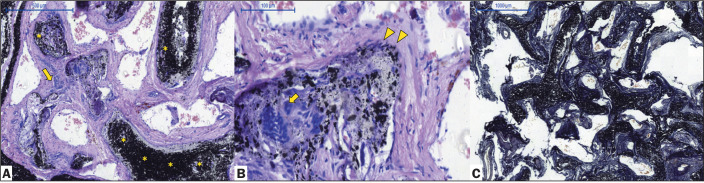
Chronic histopathological findings at 90 days after Menox LES embolization of the RM, showing intraluminal LEA (asterisks), as well as mild-to-moderate intravascular and extravascular inflammatory reactions, with a predominance of giant cells (arrows), infrequent calcifications, and some microscopic extravasation of the LEA (arrowheads, H&E staining; magnification, ×5 in A and ×20 in B), as well as elastic fibers within the vessel walls (Van Gieson staining in C; magnification, ×2)

Pig 3 (female, 33 kg) - euthanized at 90 days after the procedure: Menox LES RM embolization was performed for 30 min. There was complete occlusion of the right and middle RM, without complications during microcatheter retrieval. Angiography was performed at 90 days after the procedure, showing LEA stability and occlusion of the left and middle RM. Histopathological analysis showed mild to moderate intravascular and extravascular inflammatory reactions with a predominance of giant cells, rare calcifications, and some microscopic extravasation of the LEA (Figure 4).

**Figure 5:**
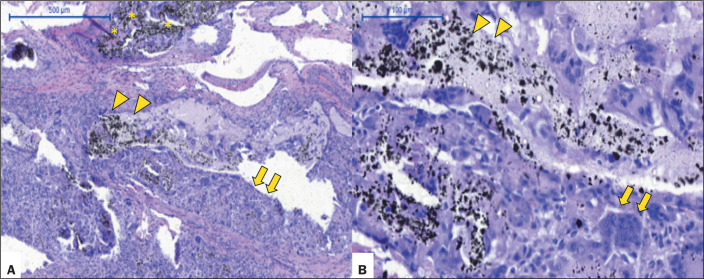
Chronic histopathological findings at 30 days after Menox LES embolization of the RM, showing intraluminal LEA (asterisks), as well as moderate-to-severe intravascular and extravascular inflammatory reactions, with a predominance of mononuclear cells and few giant cells (arrows), vessel disruption, and some microscopic extravasation of the LEA (arrowheads, H&E staining; magnification, ×5 in A and ×20 in B).

**Figure 6:**
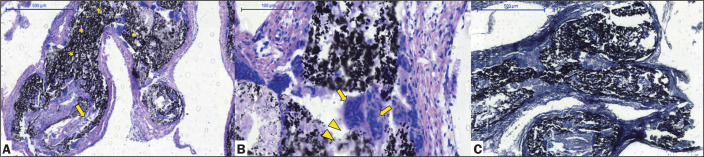
Chronic histopathological findings at 30 days after Onyx LES embolization of the RM, showing intraluminal LEA (asterisks), as well as moderate-to-severe intravascular and extravascular inflammatory reactions, with a predominance of mononuclear cells and few giant cells (arrows), together with vessel disruption and some microscopic extravasation of the LEA (arrowheads, H&E staining; magnification, ×5 in A and ×20 in B), and elastic fibers within the vessel walls (Van Gieson staining in C; magnification, ×2).

The animals in the comparison group (pigs 4 and 5) were assessed by angiography and histopathology to identify differences between Menox LES embolization and Onyx LES embolization (Figures 5 and 6).

Pig 4 (female, 32 kg) - euthanized at 30 days after the procedure: Menox LES embolization was performed on the left RM for 25 min. There was complete occlusion of the left and middle RM, with no complications during catheter retrieval. Angiography was performed at 30 days after the procedure, showing LEA stability and total occlusion of left and middle RM. Histopathological analysis showed moderate to severe intravascular and extravascular inflammatory reactions, with a predominance of mononuclear cells and few giant cells, together with vessel disruption and some microscopic extravasation of the LEA (Figure 5).

Pig 5 (female, 34 kg) - euthanized at 30 days after the procedure: Onyx LES RM embolization was performed for 23 min. There was complete occlusion of the left and middle RM, without complications during catheter retrieval. Angiography was performed at 30 days after the procedure, showing LEA stability with total occlusion of the left and middle RM. Histopathological analysis showed moderate to severe intravascular and extravascular inflammatory reactions, with a predominance of mononuclear cells and few giant cells, together with vessel disruption and some microscopic leakage of the LEA (Figure 6).

## DISCUSSION

The development of new LEAs and materials has had a major impact on the treatment of cerebral AVMs and shunts^(1,9,13-15)^. Onyx is currently the most commonly used non-adhesive LEA for intracranial endovascular embolization^(5-7)^. The EVOH-based Onyx agent allows slow polymerization and less adhesiveness, therefore providing excellent control and the ability to start and stop injections during the embolization process.

Introduced in 2018, the Menox non-adhesive EVOH copolymer allows for multiple cycles of short-lived and continuous injections. Similar to the other EVOH-based agents available, Menox advances into the vasculature with a lava-like flow pattern without any fragmentation during injections^(9)^.

In the present study, Menox and Onyx proved to have a similar technical profile. They showed equivalent radiodensity on angiography, similar obliteration rates, and required the same mean time for embolization. There were no periprocedural complications and the Menox LEA was delivered uneventfully through the microcatheter in a controlled fashion, without contralateral RM embolization or hemodynamic complications. There was complete embolization of the selected ipsilateral RM, and the microcatheter proved to be easily detached from the nidus. Clinical and histopathological outcomes were equivalent, both systems resulting in complete, stable unilateral occlusion of the RM, with minor histological changes.

Overall, Menox was delivered in a controlled fashion over a prolonged period of time with frequent pauses and with similar solidification time between the injections, ultimately resulting in predictable, targeted, and satisfactory obliteration of the nidus, with no increased risk of inadvertent embolization, or untoward extra-nidus or venous penetration in comparison with Onyx. In summary, Menox behaved in a similar fashion to Onyx, producing equivalent outcomes.

### Limitations

Despite being one of the few animal studies of Menox conducted to date, our study has some limitations. First, it was a single-center study and the technical results are limited by the individual techniques and experiences of the authors. In addition, the sample was relatively small. Furthermore, the study was not performed in a clinical setting. Therefore, the findings should be interpreted with caution because the results may not be widely applicable in general practice.

## CONCLUSION

Menox LEA proved to be safe and effective in a porcine model, promoting acute, long-lasting occlusion of the RM. In comparison with the Onyx LES, the Menox LES produced similar clinical and histopathological outcomes. Menox appears to be a safe option for the endovascular treatment of cerebral AVMs and intracranial arteriovenous shunts. Nevertheless, large multicenter studies are needed in order to confirm its clinical efficacy.
